# Efficient numerical approximation of a non-regular Fokker–Planck equation associated with first-passage time distributions

**DOI:** 10.1007/s10543-022-00914-2

**Published:** 2022-04-04

**Authors:** Udo Boehm, Sonja Cox, Gregor Gantner, Rob Stevenson

**Affiliations:** 1 Department of Psychology, University of Amsterdam, PO Box 15906, 1001 NK Amsterdam, The Netherlands; 2 Korteweg–de Vries (KdV) Institute for Mathematics, University of Amsterdam, PO Box 94248, 1090 GE Amsterdam, The Netherlands; 3 Institute of Analysis and Scientific Computing, TU Wien, Wiedner Hauptstraße 8-10, 1040 Vienna, Austria

**Keywords:** Fokker–Planck equation, Time-dependent spatial domain, Space-time variational formulation, Parameter-dependent PDE, Sparse tensor product interpolation, 30B40, 35A15, 35B65, 35K08, 60H30, 65D05, 65M12

## Abstract

In neuroscience, the distribution of a decision time is modelled by means of a one-dimensional Fokker–Planck equation with time-dependent boundaries and space-time-dependent drift. Efficient approximation of the solution to this equation is required, e.g., for model evaluation and parameter fitting. However, the prescribed boundary conditions lead to a strong singularity and thus to slow convergence of numerical approximations. In this article we demonstrate that the solution can be related to the solution of a parabolic PDE on a rectangular space-time domain with homogeneous initial and boundary conditions by transformation and subtraction of a known function. We verify that the solution of the new PDE is indeed more regular than the solution of the original PDE and proceed to discretize the new PDE using a space-time minimal residual method. We also demonstrate that the solution depends analytically on the parameters determining the boundaries as well as the drift. This justifies the use of a sparse tensor product interpolation method to approximate the PDE solution for various parameter ranges. The predicted convergence rates of the minimal residual method and that of the interpolation method are supported by numerical simulations.

## Introduction

In 1978 Ratcliff [24] introduced a model for binary decision processes based on diffusion processes. This model turned out to agree well with experimental data; Gold and Shadlen [17] provides a neurophysiological explanation for its success. Indeed, the solution $(X_t)_{t\ge 0}$ of a one-dimensional stochastic differential equation is assumed to describe the difference in activity of two competing neuron populations. At time $t = 0$, the value $X_0 = x_0\in \mathbb R$ represents the resting-state activity of the neuron populations. A decision is triggered when $(X_t)_{t\ge 0}$ first reaches one of two (possibly time-dependent) critical values $\alpha$ or $\beta$, each reflecting an outcome of the decision process.

In a typical decision experiment, scientists can only measure the decision time and outcome. Parameter fitting thus requires access to the decision time distributions, which are rarely known explicitely. Ad hoc numerical simulations are costly whence efficient simulation methods are much sought-after [15, 18].

In this article we extend and improve a simulation method introduced in [30], which is based on the Fokker–Planck equation associated to the decision time. In particular, this article may be viewed as the theoretical counterpart of our publication [3], which is aimed at the neuroscientific community.

Linking the first hitting time of a stochastic differential equation to a Fokker–Planck equation is a well-known approach that has also been applied in e.g. astrophysics [7] and cell biology [20]; for an overview see [1]. In particular, although we only consider examples arising from neuroscience, the simulation method we introduce is also relevant for other applications.

To explain the Fokker–Planck based approach consider the following stochastic differential equation:

1.1 $$\begin{aligned} dX_t^{y} = \mu (t,X_t^{y}) \,dt + \sigma \,dW_t \quad t\in {[}0,\infty ),\, X_0^{y}=y. \end{aligned}$$

Here $(W_t)_{t\in [0,\infty )}$ is a Brownian motion, $\sigma \in (0,\infty )$ is the diffusion parameter, $\mu \in C([0,\infty )\times \mathbb R)$ is the (time- and state-dependent) drift and $y\in \mathbb R$ is the initial value. Let $\alpha ,\beta \in C^1([0,\infty ))$ satisfy $\alpha \le \beta$, and for all $y\in [\alpha (0),\beta (0)]$ define the stopping times $\hat{\alpha }_{y}, \hat{\beta }_{y}$ by

1.2 $$\begin{aligned} \begin{aligned} \hat{\alpha }_{y}&:= \inf \{t \in [0,\infty ):X_t^{y} \le \alpha (t) \},\\ \hat{\beta }_{y}&:= \inf \{t \in [0,\infty ):X_t^{y} \ge \beta (t) \}. \end{aligned} \end{aligned}$$

The quantities of interest in neurophysiological decision models are the *first hitting time probabilities*: $\mathbb {P}[ \hat{\alpha }_{y} \le \min (\tau ,\hat{\beta }_{y}) ]$, where $\tau \in (0,\infty )$ and $y\in [\alpha (0),\beta (0)]$. These probabilities can be linked to the solution of a parabolic PDE. Indeed, assume $\alpha <\beta$ on $[0,\tau ]$ for some $\tau \in (0,\infty )$, set $Q := \{(t,x)\in (0,\tau )\times \mathbb R:\alpha (\tau -t)< x < \beta (\tau -t))\}$, and consider the following PDE:

1.3 $$\begin{aligned} \left\{ \begin{aligned} \partial _{t} F(t,x)&= \tfrac{\sigma ^2}{2}\partial ^2_{x} F(t,x) + \mu (\tau -t,x) \partial _{x} F(t,x)&\quad (t,x)\in Q_{\tau },\\ F(t,\alpha (\tau -t))&=1, \quad F(t,\beta (\tau -t))=0&\quad t \in (0,\tau ),\\ F(0, x)&=0&\quad x \in (\alpha (\tau ), \beta (\tau )). \end{aligned} \right. \end{aligned}$$

Under some additional regularity assumptions on $\alpha$, $\beta$, and $\mu$ it can be shown that a solution to (1.3) exists and satisfies

1.4 $$\begin{aligned} \mathbb {P}[ \hat{\alpha }_{y} \le \min (\tau ,\hat{\beta }_{y}) ] = F(\tau ,y),\quad \alpha (0)\le y \le \beta (0). \end{aligned}$$

(see [30, Appendix A] for the case that $\alpha$ and $\beta$ are constant and $\mu$ does not depend on time or [23, Chapter 7] for general Fokker–Planck equations, also known in this setting as a backward Kolmogorov equation).

In [30], a Crank–Nicolson method is used to approximate solutions to (1.3) in the case that $\alpha$, $\beta$, and $\mu$ are constant. One advantage of this setting is that one only needs to solve a single PDE of type (1.3) in order to obtain the first hitting time probabilities $\mathbb {P}[ \hat{\alpha }_{y} \le \min (t,\hat{\beta }_{y}) ]$ for all $t\in [0,\tau ],\, y\in [\alpha (0),\beta (0)]$. However, due to the fact that *F* is discontinuous at $(t,x)=(0,\alpha (\tau ))$, no proof of convergence of the Crank–Nicolson for decreasing step-sizes seems available. At best, reduced rates are to be expected. Moreover, various authors have argued that time-dependent boundaries $\alpha$ and $\beta$ and space-time-dependent drift $\mu$ provide a more realistic model for decision processes, for an overview see [18, 25].

In this article we *extend* [30] to include diffusion models with time-dependent boundaries and non-constant drift. We *improve* the efficiency of the numerical simulation by not approximating the solution *F* to (1.3) directly, instead, we approximate the solution to a parabolic PDE on a rectangular domain with homogeneous initial and boundary conditions constructed such that its difference with *F* (transformed to the same rectangular domain) is a function for which a rapidly converging series expansion is known.

More specifically, in Section 2 we demonstrate that if $\alpha ,\beta$ are once continuously differentiable, then (1.3) can be transformed into a parabolic PDE on a rectangular domain with a space-time-dependent drift. Next, in Section 3 we demonstrate that by subtracting a known, discontinuous function, we obtain a parabolic PDE with homogeneous boundary conditions, see (3.1) below. We analyze the regularity of the solution *e* to this equation and verify that it is indeed smoother than *F*, see Corollary 3.1 and Theorem 3.1.

In Section 4 we apply a minimal residual method [2, 28, 29] to approximate the solution *e* to (3.1). This method is known to give quasi-best approximations from the selected trial space in the norm on a natural solution space being the intersection of two Bochner spaces. Taking as trial space the space of continuous piecewise bilinears with respect to a uniform partition of the space-time cylinder into rectangles with mesh width *h*, in Theorem 4.1 the optimal error bound of order *h* is shown for the solution *e* to (3.1).

In Section 5 we consider the situation that $\mu$, $\alpha$, and $\beta$ can be parametrized analytically and verify that in this case the corresponding solution *e* to  (3.1) (transformed onto the unit square) depends analytically on these parameters as well as on the final time $\tau$, see Theorem 5.1. This justifies the use of a sparse tensor-product interpolation [22] to determine the solution *e* to  (3.1) efficiently for multiple end-time and parameter values. Finally, in Section 6 we provide numerical simulations for three different decision models taken from the neurophysiological literature.

In our parallel publication [3] mentioned above, we provide further numerical experiments and code. There, we apply the Crank–Nicolson method (without giving any error analysis) to approximate the solution *e* to (3.1). In the examples we consider it appears that the Crank–Nicolson method leads to similar convergence as the minimal residual method. Although we only provide a rigorous error analysis for the minimal residual method, Crank–Nicolson may be preferred in practice as it is easier to implement. We refer to [3] for further details.

### Notation

In this work, by $C \lesssim D$ we mean that *C* can be bounded by a multiple of *D*, independently of parameters which *C* and *D* may depend on. Obviously, $C \gtrsim D$ is defined as $D \lesssim C$, and $C\eqsim D$ as $C\lesssim D$ and $C \gtrsim D$.

For normed linear spaces *E* and *F*, by $\mathcal L(E,F)$ we denote the normed linear space of bounded linear mappings $E \rightarrow F$, and by $\mathcal L_{\mathrm {iso}}(E,F)$ its subset of boundedly invertible linear mappings $E \rightarrow F$.

## Transforming the Fokker–Planck equation to a rectangular space-time domain

In this section we demonstrate that (1.3) can be transformed into a PDE on a rectangular space-time domain, see (2.3) below. The PDE in (2.3) below forms the starting point for the remainder of this article, which is why we use tildes in (2.1) below to distinguish the variables and coefficients of the non-transformed equation from those in (2.3). Indeed, let $\widetilde{T} \in (0,\infty ]$, assume $a,b \in C^{1}([0,\widetilde{T}))$ satisfy $a(\tilde{t})< b(\tilde{t})$ for all $\tilde{t}\in [0,\widetilde{T})$, set $\widetilde{Q} := \{ (\tilde{t},\tilde{x})\in (0,\widetilde{T})\times \mathbb R:a(\tilde{t})< \tilde{x} < b(\tilde{t})\}$, let $\tilde{v} \in L_\infty (\widetilde{Q})$, and consider the following parabolic initial- and boundary value problem:

2.1 $$\begin{aligned} \left\{ \begin{aligned} \partial _{\tilde{t}} \tilde{u}(\tilde{t}, \tilde{x})&= \partial ^2_{\tilde{x}} \tilde{u}(\tilde{t}, \tilde{x}) +\tilde{v}(\tilde{t}, \tilde{x}) \partial _{\tilde{x}} \tilde{u}(\tilde{t}, \tilde{x})&\quad (\tilde{t}, \tilde{x}) \in \widetilde{Q},\\ \tilde{u}(\tilde{t},a(\tilde{t}))&=1, \quad \tilde{u}(\tilde{t},b(\tilde{t}))=0&\quad \tilde{t} \in (0,\widetilde{T}),\\ \tilde{u}(0, \tilde{x})&=0&\quad \tilde{x} \in (a(0), b(0)). \end{aligned} \right. \end{aligned}$$

Note that this is (1.3) with $\tilde{u}(\tilde{t},\tilde{x}) = F(\frac{2\tilde{t}}{\sigma ^2},\tilde{x})$, $\widetilde{T}=\frac{\sigma ^2 \tau }{2}$, $a(\tilde{t})=\alpha (\frac{2}{\sigma ^2}(\widetilde{T}-\tilde{t}))$, $b(\tilde{t})=\beta (\frac{2}{\sigma ^2}(\widetilde{T}-\tilde{t}))$, $\tilde{v}(\tilde{t},\tilde{x})=\frac{2}{\sigma ^2}\mu (\frac{2}{\sigma ^2}(\widetilde{T}-\tilde{t}),\tilde{x} )$.

Now, set $T:=\int _{0}^{\tilde{T}} |b(\tilde{s})-a(\tilde{s})|^{-2} \,d\tilde{s}$ (where possibly $T=\infty$) and define $\theta :[0,T) \rightarrow [0,\tilde{T})$ by $\theta (t) = \sup \left\{ \tilde{r}\in [0,\widetilde{T}) :\int _{0}^{\tilde{r}} |b(\tilde{s})-a(\tilde{s})|^{-2} \,d\tilde{s} \le t \right\}$, then $\theta$ is a bijection and $\theta ^{-1}(\tilde{t}) = \int _{0}^{\tilde{t}} |b(\tilde{s})-a(\tilde{s})|^{-2} \,d\tilde{s}$. In particular, from $t=\theta ^{-1}(\theta (t))$ we obtain that $\theta$ satisfies the following ODE

2.2 $$\begin{aligned} \theta '(t) = \big (b(\theta (t)) - a(\theta (t))\big )^2,\quad \theta (0)=0. \end{aligned}$$

With

$$\begin{aligned} \Omega :=(0,1), \end{aligned}$$

and $\xi :[0,\widetilde{T})\times \overline{\Omega } \rightarrow \mathbb R$ defined by

$$\begin{aligned} \xi (\tilde{t},x) := (1-x) a(\tilde{t})+x b(\tilde{t}), \end{aligned}$$

we have that

$$\begin{aligned} {[}0,T) \times \Omega \rightarrow \widetilde{Q}:(t,x) \mapsto (\theta (t),\xi (\theta (t),x)) \end{aligned}$$

is a bijection with inverse

$$\begin{aligned} (\tilde{t},\tilde{x}) \mapsto \Big ( \theta ^{-1}(\tilde{t}), \frac{\tilde{x}-a(\tilde{t})}{b(\tilde{t})-a(\tilde{t})} \Big ). \end{aligned}$$

Defining $$u,v:[0,T)\times \overline{\Omega } \rightarrow \mathbb R$$ by

$$\begin{aligned} u(t,x)&:= \tilde{u}(\theta (t),\xi (\theta (t),x)),\\ v(t,x)&:= (b(\theta (t))-a(\theta (t))) \big [\tilde{v}(\theta (t),\xi (\theta (t),x))+(1-x)a'(\theta (t)))+x b'(\theta (t))\big ], \end{aligned}$$

we have $u(t,0)=1$, $u(t,1)=0$ ($t \in (0,T)$), and $u(0,x)=0$ ($x \in \Omega$). Moreover, for $(t,x) \in (0,T)\times \Omega$, one has

$$\begin{aligned} \partial _{x} u(t,x)&= (b(\theta (t))-a(\theta (t))) \partial _{\tilde{x}} \tilde{u}(\theta (t),\xi (\theta (t),x)),\\ \partial ^2_{x} u(t,x)&= (b(\theta (t))-a(\theta (t)))^2 \partial ^2_{\tilde{x}} \tilde{u}(\theta (t),\xi (\theta (t),x)), \end{aligned}$$

and

$$\begin{aligned} \partial _t u(t,x)&= \theta '(t)\big \{\partial _{\tilde{t}} \tilde{u}(\theta (t), \xi (\theta (t),x))+\partial _{\tilde{t}} \xi (\theta (t),x)\partial _{\tilde{x}} \tilde{u}(\theta (t),\xi (\theta (t),x))\big \}\\&= (b(\theta (t))-a(\theta (t)))^2 \Big \{\partial _{\tilde{t}} \tilde{u}(\theta (t), \xi (\theta (t),x))\\&\quad +\,\big [(1-x)a'(\theta (t))+x b'(\theta (t))\big ] \partial _{\tilde{x}} \tilde{u}(\theta (t),\xi (\theta (t),x))\Big \}\\&= (b(\theta (t))-a(\theta (t)))^2 \Big \{\partial ^2_{\tilde{x}} \tilde{u}(\theta (t), \xi (\theta (t),x))\\&\quad +\,\big [\tilde{v}(\theta (t), \xi (\theta (t),x))+(1-x)a'(\theta (t))+x b'(\theta (t))\big ] \partial _{\tilde{x}} \tilde{u}(\theta (t),\xi (\theta (t),x))\Big \}\\&=\partial ^2_{x} u(t,x)+ v(t,x) \partial _{x} u(t,x). \end{aligned}$$

In other words, with

$$\begin{aligned} I:=(0,T), \end{aligned}$$

(2.1) is equivalent to finding $u=u(v)$ that solves

2.3 $$\begin{aligned} \left\{ \begin{aligned} \partial _{t} u(t, x)&= \partial ^2_{x} u(t, x) +v(t, x) \partial _{x} u(t, x)&\quad (t, x) \in I \times \Omega ,\\ u(t,0)&=1, \quad u(t,1)=0&\quad t \in I,\\ u(0, x)&=0&\quad x \in \Omega . \end{aligned} \right. \end{aligned}$$

To be able to numerically solve (2.3), we assume from now on that $T <\infty$.

### Example 2.1

Bowman, Kording, and Gottfried [4] suggested collapsing boundaries, i.e., in (1.3) they take $\alpha (t):= \frac{\beta _0 t}{2T_0}$ and $\beta (t) := \beta _0(1-\frac{t}{2T_0})$ for some fixed parameters $\beta _0, T_0 \in (0,\infty )$. Translating this to the setting of (2.1), this leads to $a(\tilde{t}):=\frac{ \beta _0 (\widetilde{T}-\tilde{t})}{\sigma ^2 T_0}$ and $b(\tilde{t}):= \beta _0(1-\frac{\widetilde{T}-\tilde{t}}{\sigma ^2 T_0})$ (note that it only makes sense to consider $\widetilde{T}\in (0,\frac{\sigma ^2 T_0}{2} )$ in this setting). Note that it is easier to first determine $\theta ^{-1}(\tilde{t}) = \int _{0}^{\tilde{t}} |b(\tilde{s}) - a(\tilde{s})|^{-2} \,d\tilde{s}$ and then determine $T=\theta ^{-1}(\tilde{T})$ and $\theta = (\theta ^{-1})^{-1}$. Indeed, $\theta ^{-1}(\tilde{t}) = \frac{\sigma ^4 T_0^2 \tilde{t}}{\beta _0^2(\sigma ^2 T_0 -2\widetilde{T})(\sigma ^2 T_0 -2\widetilde{T} +2\tilde{t})}$ and thus $T = \frac{\sigma ^2 T_0 \widetilde{T}}{\beta _0^2(\sigma ^2 T_0 - 2\widetilde{T})}$ and

$$\begin{aligned} \theta (t) = \frac{ \beta _0^2(\sigma ^2 T_0- 2\widetilde{T})^2 t }{ \sigma ^4 T_0^2 - 2\beta _0^2(\sigma ^2 T_0- 2 \widetilde{T})t },\quad t\in [0,T). \end{aligned}$$

By observing that $(1-x)a'(\theta (t))+x b'(\theta (t))=\frac{(2x-1) \beta _0}{\sigma ^2 T_0}$, and $b(\theta (t))-a(\theta (t))$ $= \frac{b_0(1-2\sigma ^2 T_0 \widetilde{T})}{\sigma ^4 T_0^2-2 \beta _0^2(\sigma ^2 T_0-2 \widetilde{T}) t}$, one obtains *v* in terms of $\tilde{v}$.

## Regularity of the Fokker–Planck equation

Let *u*(*v*) denote the solution to (2.3) for some given drift function *v*. Due to the discontinuity between boundary and initial data, it is clear that *u*(*v*) is discontinuous at the corner $(t,x)=(0,0)$. This reduces the rate of convergence of standard numerical methods and makes it difficult to provide a theoretical bound on the convergence rate. However, for *constant drift* *v*, a rapidly converging series expansion of *u*(*v*) is known ([16]), which allows to efficiently approximate *u*(*v*) within any given positive tolerance. Knowing this, our approach to approximate *u*(*v*) for *variable* $v \in C(\overline{I \times \Omega })$ is to *approximate the difference*

$$\begin{aligned} e=e(v)=u(v)-u(v_{0}), \text { where } v_{0}:=v(0,0). \end{aligned}$$

This function *e*(*v*) solves

3.1 $$\begin{aligned} \left\{ \begin{aligned} \partial _t e(t, x)&= \partial ^2_x e(t, x) \!+\! v(t, x) \partial _x e(t, x)\!+\! (v(t, x)-v_{0}) \partial _x u(v_{0})&\quad (t, x) \in I\times \Omega ,\\ e(t,0)&=0, \quad e(t,1)=0&\quad t \in I,\\ e(0, x)&=0&\quad x \in \Omega , \end{aligned} \right. \end{aligned}$$

which we solve approximately with a numerical method. To derive a priori bounds for the approximation error, we analyze the smoothness of *e*(*v*), see Section 3.3. In particular, under additional smoothness conditions on *v*, and using that $(v-v_{0})(0,0)=0$, we show that

$$\begin{aligned} e(v) \text { is more smooth than } u(v_{0}), \text { and thus than }u(v), \end{aligned}$$

which shows the benefit of applying the numerical method to (3.1) instead of directly to (2.3).

It turns out that for any *v* the smoothness of *u*(*v*) is determined by that of the solution $u_H$ of the heat equation on $(0,\infty ) \times \mathbb R$ that is 0 at $t=0$ and 1 at $x=0$. Its smoothness is the topic of the next subsection.

### The heat kernel

The function

$$\begin{aligned} H(t,x):=\frac{1}{2\sqrt{\pi t}} e^{-\frac{x^2}{4t}} \end{aligned}$$

is the heat kernel. It satisfies

$$\begin{aligned} \begin{aligned} \partial _t H(t,x)&=\partial ^2_x H(t,x)&(t, x) \in (0,\infty ) \times \mathbb R,\\ \lim _{t \downarrow 0} \int _\mathbb RH(t,x) \phi (x) \,d x&=\phi (0)&\text {for all } \phi \in \mathcal {D}(\mathbb R), \end{aligned} \end{aligned}$$

the latter being the space of *test functions*.

Following [10, Ex. 2.14] and [14], for $(t, x) \in (0,\infty ) \times \mathbb R$ we define

$$\begin{aligned} u_H(t,x):=2 \int _x^\infty H(t,y)\,dy={\textstyle \frac{2}{\sqrt{\pi }}} \int _{\frac{x}{2\sqrt{t}}}^\infty e^{-s^2}\,ds={{\,\mathrm{Erfc}\,}}({\textstyle \frac{x}{2\sqrt{t}}}). \end{aligned}$$

Knowing that $\int _{0}^\infty {\textstyle \frac{1}{\sqrt{\pi t}}} e^{-\frac{y^2}{4t}} \,dy=1$, and ${\displaystyle \lim _{t \downarrow 0}} \int _{x}^\infty {\textstyle \frac{1}{\sqrt{\pi t}}} e^{-\frac{y^2}{4t}} \,dy=0$ for $x>0$, we have

$$\begin{aligned} \left\{ \begin{aligned}{2} \partial _t u_H(t,x)&=\partial ^2_x u_H(t,x)&\quad (t, x) \in (0,\infty ) \times \mathbb R,\\ u_H(t,0)&=1&\quad t>0,\\ u_H(0,x):=\lim _{t \downarrow 0} u_H(t,x)&=0&\quad x>0. \end{aligned} \right. \end{aligned}$$

The following lemma turns out to be handy to analyze the smoothness of $u_H$ restricted to $I \times \Omega$.

#### Lemma 3.1

For $p>0$, $\alpha ,\beta \in \mathbb R$, it holds that $\int _0^T \int _0^1 |t^\alpha x^\beta e^{-\frac{x^2}{4t}}|^p \,dx\,dt<\infty$ if and only if $p \beta >-1$ and $p(2\alpha +\beta ) >-3$.

#### Proof

The mapping

$$\begin{aligned} \Phi :\big \{(\lambda ,x) \in (0,\infty )\times (0,1):x<2 \sqrt{\lambda T}\big \} \rightarrow (0,T)\times (0,1):(\lambda ,x) \mapsto \big (\frac{x^2}{4\lambda },x\big ) \end{aligned}$$

is a diffeomorphism, and $|D\Phi (\lambda ,x)|=\frac{x^2}{4 \lambda ^2}$. One obtains

$$\begin{aligned} \int _0^T\int _0^1 |t^\alpha x^\beta e^{-\frac{x^2}{4t}}|^p \,dx\,dt= \int _0^\infty \int _0^{\min (1,2 \sqrt{\lambda T})} \big (\frac{x^2}{4 \lambda }\big )^{\alpha p} x^{\beta p} e^{-p \lambda }\frac{x^2}{4 \lambda ^2} \,dx\,d\lambda \\ = 4^{-\alpha p -1} \int _0^\infty \lambda ^{-\alpha p -2} e^{-p \lambda } \int _0^{\min (1,2 \sqrt{\lambda T})} x^{2 \alpha p+\beta p+2}\,dx \,d \lambda . \end{aligned}$$

The integral over *x* is finite if and only if $p(2\alpha +\beta ) >-3$, and if so, the expression is equal to

$$\begin{aligned} {\textstyle \frac{4^{-\alpha p -1}}{2 \alpha p+\beta p+3}} \Big [ (2\sqrt{T})^{2\alpha p +\beta p+3} \int _0^\frac{1}{4T} \lambda ^{(\beta p-1)/2} e^{-p \lambda } d \lambda + \int _\frac{1}{4T}^\infty \lambda ^{-\alpha p -2} e^{-p \lambda } \,d\lambda \Big ] \end{aligned}$$

with the first integral being finite if and only if $p \beta >-1$. $\square$

Following [31], we analyze the regularity of the solutions *u*(*v*) and *e*(*v*) of the parabolic problems  (2.3) and (3.1), respectively, in (intersections of) Bochner spaces. In particular, the space $L_2(I;H^1(\Omega )) \cap H^1(I;H^{-1}(\Omega ))$ plays an important role in this and following sections. For the precise definition of this space and some properties we refer to [31, Chapter 25]. With $H^1_{0,\{0\}}(I)$ denoting the closure in $H^1(I)$ of the functions in $C^\infty (I) \cap H^1(I)$ that vanish at 0, we have the following result concerning the smoothness of $u_H$ restricted to $I \times \Omega$.

#### Corollary 3.1

$u_H \in L_2(I;H^1(\Omega )) \cap H^1_{0,\{0\}}(I;H^{-1}(\Omega ))$, but $u_H \not \in H^1_{0,\{0\}}(I;L_2(\Omega ))$ and $u_H \not \in L_2(I;H^2(\Omega ))$. Furthermore, $t \partial _t \partial _x u_H, x \partial ^2_x u_H, t \partial ^2_x u_H \in L_2(I \times \Omega )$, and $x \partial _t \partial _x u_H \in L_2(I;H^{-1}(\Omega ))$.

#### Proof

By applications of Lemma 3.1, we infer that $\partial _x u_H=-2H \in L_2(I \times \Omega )$, and that $\partial _t u_H(t,x)=\frac{1}{2\sqrt{\pi }} x t^{-\frac{3}{2}} e^{-\frac{x^2}{4t}} \not \in L_2(I \times \Omega )$. This yields $u_H \in L_2(I;H^1(\Omega ))$ and $u_H \not \in H_{0,\{0\}}^1(I;L_2(\Omega ))$.

If $\partial _x F=f$, then $f \in L_2(I;H^{-1}(\Omega ))$ if and only if $F \in L_2(I \times \Omega )$. We have $\int _{-\infty }^x \partial _t u_H(t,y)\,dy=-\frac{t^{-\frac{1}{2}}}{\sqrt{\pi }}e^{-\frac{x^2}{4t}} \in L_2(I \times \Omega )$, so indeed $u_H \in H^1_{0,\{0\}}(I;H^{-1}(\Omega ))$.

It holds

$$\begin{aligned} \partial _x^2 u_H(t,x)=-2\partial _x H(t,x)=\frac{1}{2 \sqrt{\pi }} x t^{-\frac{3}{2}} e^{-\frac{x^2}{4t}} \not \in L_2(I \times \Omega ), \end{aligned}$$

or $$u_H \not \in L_2(I;H^2(\Omega ))$$, but $$ x \partial ^2_x u_H, t \partial ^2_x u_H \in L_2(I \times \Omega )$$.

We have $\partial _x\partial _t u_H=(t^{-\frac{3}{2}}-\frac{1}{2} x^2 t^{-\frac{5}{2}}) \frac{e^{-\frac{x^2}{4t}}}{2 \sqrt{\pi }}$, so $t \partial _x\partial _t u_H \in L_2(I \times \Omega )$. Proving that $x \partial _x\partial _t u_H \in L_2(I;H^{-1}(\Omega ))$ amounts to proving $x t^{-\frac{3}{2}}e^{-\frac{x^2}{4t}},\,t^{-\frac{5}{2}}x^3e^{-\frac{x^2}{4t}} \in L_2(I;H^{-1}(\Omega ))$, i.e., proving that $t^{-\frac{3}{2}} \int _{-\infty }^x y e^{-\frac{y^2}{4t}}dy,\,t^{-\frac{5}{2}}\int _{-\infty }^x y^3e^{-\frac{y^2}{4t}} dy \in L_2(I \times \Omega )$. The first function equals $-2t^{-\frac{1}{2}}e^{-\frac{x^2}{4t}}$, which is in $L_2(I \times \Omega )$, and the second function equals $-8t^{-\frac{1}{2}} e^{-\frac{x^2}{4t}} -2t^{-\frac{3}{2}} x^2 e^{-\frac{x^2}{4t}}$, which is also in $L_2(I \times \Omega )$. $\square$

Finally in this subsection, notice that from $\partial _t u_H(t,x)=\frac{1}{2\sqrt{\pi }} x t^{-\frac{3}{2}} e^{-\frac{x^2}{4t}}$, it follows that for any $x>0$ and $k \in \mathbb N_0$,

3.2 $$\begin{aligned} \lim _{t \downarrow 0}\partial ^k_t u_H(t,x)=0. \end{aligned}$$

### Regularity of the parabolic problem with homogeneous initial and boundary conditions

Knowing that *e*(*v*) is the solution of the parabolic problem (3.1) that has homogeneous initial and boundary conditions, we study the regularity of such a problem.

Given functions $v\in L_{\infty }(I\times \Omega )$ and $f\in L_2(I;H^{-1}(\Omega ))$, let *w* solve

3.3 $$\begin{aligned} \left\{ \begin{aligned} \partial _t w(t, x)&= \partial ^2_x w(t, x) +v(t, x) \partial _x w(t, x)+f(t,x)&\quad (t, x) \in I\times \Omega ,\\ w(t,0)&=0, \quad w(t,1)=0&\quad t \in I,\\ w(0, x)&=0&\quad x \in \Omega , \end{aligned} \right. \end{aligned}$$

where the spatial differential operators at the right-hand side should be interpreted in a weak sense, i.e., $((\partial _x^2 +v\partial _x)\eta )(\zeta ):=\int _D -\partial _x \eta \partial _x \zeta + v\partial _x \eta \,\zeta \,dx$. It is well-known that

3.4 $$\begin{aligned} L(v):=w \mapsto f \in \mathcal L_{\mathrm {iso}}(L_2(I;H^1_0(\Omega )) \cap H^1_{0,\{0\}}(I;H^{-1}(\Omega )),L_2(I;H^{-1}(\Omega ))) \end{aligned}$$

(see, e.g., [31, Thm. 26.1]). Under additional smoothness conditions on the right-hand side *f* beyond being in $L_2(I;H^{-1}(\Omega ))$, additional smoothness of the solution *w* can be demonstrated:

#### Proposition 3.1

- If $$v \in W^1_\infty (I \times \Omega )$$, then
  $$\begin{aligned} L(v)^{-1} \in \mathcal L\Big (&L_2(I;H^1(\Omega )) \cap H^1(I;H^{-1}(\Omega )),\\&H^1_{0,\{0\}}(I;H^1_0(\Omega ))\cap H^2(I;H^{-1}(\Omega )) \cap L_2(I;H^3(\Omega )) \Big ). \end{aligned}$$
- If $$v \in L_\infty (I\times \Omega )$$, then
  $$\begin{aligned} L(v)^{-1} \in \mathcal L\Big (L_2(I \times \Omega ),L_2(I;H^2(\Omega )) \cap H^1_{0,\{0\}}(I;L_2(\Omega ))\Big ), \end{aligned}$$

#### Proof

a) If $f \in L_2(I;H^1(\Omega )) \cap H^1(I;H^{-1}(\Omega ))$, then also $f \in H^1(I;H^{-1}(\Omega ))$, and $f(0,\cdot ) \in L_2(\Omega )$ with $\Vert f(0,\cdot )\Vert _{L_2(\Omega )} \lesssim \Vert f\Vert _{L_2(I;H^1(\Omega ))}$ $+$ $\Vert f\Vert _{H^1(I;H^{-1}(\Omega ))}$ (see, e.g.,  [31, Thm. 25.5]). As shown in [31, Thm. 27.2 and its proof], from the last two properties of *f*, and $v \in W_\infty ^1(I;L_\infty (\Omega ))$, one has $w=L(v)^{-1} f \in H^1_{0,\{0\}}(I;H^1_0(\Omega ))\cap H^2(I;H^{-1}(\Omega ))$ with

$$\begin{aligned} \Vert w \Vert _{H^1_{0,\{0\}}(I;H^1_0(\Omega ))\cap H^2(I;H^{-1}(\Omega ))} \lesssim \Vert f\Vert _{H^1(I;H^{-1}(\Omega ))}+\Vert f(0,\cdot )\Vert _{L_2(\Omega )}. \end{aligned}$$

To show the spatial regularity, i.e., $w \in L_2(I;H^3(\Omega ))$, given a constant $\lambda$, we define $w_\lambda (t,\cdot )=w(t,\cdot ) e^{-\lambda t}$, $f_\lambda (t,\cdot )=f(t,\cdot ) e^{-\lambda t}$. One infers that

3.5 $$\begin{aligned} (-\partial _x^2 -v \partial _x+\lambda )w_\lambda =\underbrace{f_\lambda -\partial _t w_\lambda }_{g_\lambda :=} \,\,\,\text { on } I \times \Omega ,\quad w_\lambda (\cdot ,0)=0=w_\lambda (\cdot ,1) \,\,\, \text { on } I, \end{aligned}$$

where, as before, the spatial differential operators should be interpreted in a weak sense. Using that

$$\begin{aligned} \left| \int _I \int _D v (\partial _x w_\lambda )\,w_\lambda \,dx\,dt \right| \le \Vert v\Vert _{L_\infty (I \times \Omega )} \Vert \partial _x w_\lambda \Vert _{L_2(I\times \Omega )} \Vert w_\lambda \Vert _{L_2(I \times \Omega )} \end{aligned}$$

and Young’s inequality, one infers that for $\lambda > \frac{1}{4}\Vert v\Vert ^2_{L_\infty (I \times \Omega )}$ the bilinear form defined by the left-hand side of (3.5) is bounded and *coercive* on $L_2(I;H^1_0(\Omega )) \times L_2(I;H^1_0(\Omega ))$. Thus for $\lambda > \frac{1}{4}\Vert v\Vert ^2_{L_\infty (I \times \Omega )}$ we have

$$\begin{aligned} A(v,\lambda ):=w_\lambda \mapsto g_\lambda \in \mathcal L_{\mathrm {iso}}(L_2(I;H^1_0(\Omega )),L_2(I;H^{-1}(\Omega ))). \end{aligned}$$

Realizing that $\Vert \cdot \Vert _{H^{k+2}(\Omega )}^2=\Vert \frac{\mathrm {d}^k}{\mathrm {d}x^k} \frac{\mathrm {d}^2}{\mathrm {d} x^2}\cdot \Vert _{L_2(\Omega )}^2+\Vert \cdot \Vert _{H^{k+1}(\Omega )}^2$, an induction and tensor product argument shows $A(0,0)^{-1} \in \mathcal L(L_2(I;H^k(\Omega )),L_2(I;H^{k+2}(\Omega )))$ for any $k \in \mathbb N_0$. Writing

$$\begin{aligned} A(v,\lambda )^{-1}-A(0,0)^{-1}=A(0,0)^{-1}(v \partial _x-\lambda \mathrm {Id})A(v,\lambda )^{-1}, \end{aligned}$$

and using that $v \partial _x \in \mathcal L(L_2(I;H^1(\Omega )), L_2(I;L_2(\Omega ))$ by $v \in L_\infty (I\times \Omega )$, one verifies that $A(v,\lambda )^{-1} \in \mathcal L(L_2(I \times \Omega ),L_2(I;H^{2}(\Omega )))$. Repeating the argument, now using that $v \partial _x \in \mathcal L(L_2(I;H^2(\Omega )), L_2(I;H^1(\Omega ))$ by $v \in L_\infty (I;W_\infty ^1( \Omega ))$, one has $A(v,\lambda )^{-1} \in \mathcal L(L_2(I;H^1(\Omega )),L_2(I;H^{3}(\Omega )))$. Knowing that $f_\lambda -\partial _t w_\lambda$ $\in$ $L_2(I;H^1(\Omega ))$ with $\Vert f_\lambda -\partial _t w_\lambda \Vert _{L_2(I;H^1(\Omega ))} \lesssim \Vert f\Vert _{L_2(I;H^1(\Omega ))}+\Vert f\Vert _{H^1(I;H^{-1}(\Omega ))}$, one infers that $w_\lambda$ and thus $w \in L_2(I;H^{3}(\Omega ))$, and moreover $\Vert w \Vert _{L_2(I;H^{3}(\Omega ))} \lesssim \Vert f\Vert _{L_2(I;H^1(\Omega ))}+\Vert f\Vert _{H^1(I;H^{-1}(\Omega ))}$.

b) Similar to Part a), it suffices to show that

$$\begin{aligned} L(v,\lambda )^{-1}:=f_\lambda \mapsto w_\lambda \in \mathcal L\Big (L_2(I \times \Omega ),L_2(I;H^2(\Omega )) \cap H^1_{0,\{0\}}(I;L_2(\Omega ))\Big ). \end{aligned}$$

Knowing that $L(v,\lambda )^{-1} \in \mathcal L\big (L_2(I;H^{-1}(\Omega )), L_2(I;H^1_0(\Omega )) \cap H^1_{0,\{0\}}(I;H^{-1}(\Omega ))\big )$, and $L(v,\lambda )-L(0,0) = -v \partial _x+\lambda \mathrm {Id}\in \mathcal L\big (L_2(I;H^1_0(\Omega )),L_2(I\times \Omega )\big )$, the proof is completed by $L(v,\lambda )^{-1}-L(0,0)^{-1}=L(0,0)^{-1}(L(0,0)-L(v,\lambda ))L(v,\lambda )^{-1}$ and the *maximal regularity* result

$$\begin{aligned} L(0,0)^{-1} \in \mathcal L\Big (L_2(I \times \Omega ),L_2(I;H^2(\Omega )) \cap H^1_{0,\{0\}}(I;L_2(\Omega ))\Big ) \end{aligned}$$

from, e.g., [11, 12]. $$\square $$

### The regularity of $e(v)=u(v)-u(v_{0})$

Recall that $u_H$ denotes the solution of the heat equation studied in Section 3.1, that *u*(*v*) denotes the solution to (3.1) for given $v\in C(\overline{I\times \Omega })$, and $v_0:=v(0,0)$. Since *e*(*v*) solves (3.1), i.e., *e*(*v*) is the solution *w* of (3.3) for forcing function *f* given by

3.6 $$\begin{aligned} \begin{aligned}&(v-v_{0})\partial _x u(v_{0}) \\&\quad = (v-v_{0})\partial _x (u(v_{0})-u(0))+ (v-v_{0})\partial _x (u(0)-u_H)+ (v-v_{0})\partial _x u_H, \end{aligned} \end{aligned}$$

in view of the regularity results proven in Proposition 3.1, we establish smoothness of *e*(*v*) by demonstrating smoothness of each of the three terms at the right-hand side of (3.6).

#### Lemma 3.2

It holds that

$$\begin{aligned} u(0)-u_H \in H^1_{0,\{0\}}(I;H^1_0(\Omega ))\cap H^2(I;H^{-1}(\Omega )) \cap L_2(I;H^3(\Omega )). \end{aligned}$$

#### Proof

The function $w(t,x):=u(0)(t,x)-(u_H(t,x)-xu_H(t,1))$ satisfies the homogeneous initial and boundary conditions from (3.3), and $\partial _t w(t,x)=\partial _x^2 w(t,x) +x \partial _t u_H(t,1)$. By (3.2) we have $(t,x) \mapsto x \partial _t u_H(t,1) \in L_2(I;H^1(\Omega )) \cap H^1(I;H^{-1}(\Omega ))$, so that Proposition 3.1a) for $v=0$ and $f(t,x)= x\partial _t u_H(t,1)$ shows that

$$\begin{aligned} w \in H^1_{0,\{0\}}(I;H^1_0(\Omega ))\cap H^2(I;H^{-1}(\Omega )) \cap L_2(I;H^3(\Omega )). \end{aligned}$$

Because, again by (3.2), $(t,x) \mapsto xu_H(t,1)$ is in the same space, the proof is completed. $\square$

#### Lemma 3.3

For *any* $$v_0\in \mathbb R$$, $$u(v_0) -u(0) \in L_2(I;H^2(\Omega )) \cap H^1_{0,\{0\}}(I;L_2(\Omega ))$$.

#### Proof

The function $w:=u(v_0)-u(0)$ satisfies the homogeneous initial- and boundary conditions from (3.3), and $\partial _t w(t,x)=\partial _x^2 w(t,x) +v_0 \partial _x w-v_0 \partial _x u(0)$. From $\partial _x u(0) \in L_2(I \times \Omega )$ by Corollary 3.1 and Lemma 3.2, an application of Proposition 3.1b) for $v = v_0$ and $f=-v_0 \partial _x u(0)$ completes the proof. $\square$

#### Lemma 3.4

If $$v \in W^1_\infty (I \times \Omega ) \cap L_\infty (I;W^2_\infty (\Omega ))$$, then

$$\begin{aligned} (v-v_{0}) \partial _x u_H \in L_2(I;H^1(\Omega )) \cap H^1(I;H^{-1}(\Omega )). \end{aligned}$$

#### Proof

Abbreviate $g:=(v-v_{0}) \partial _x u_H$. Throughout the proof, we use the estimates for $u_H$ proven in Corollary 3.1.

We start with proving $\partial _t g =(\partial _t v) \partial _x u_H +(v-v_{0} ) \partial _t \partial _x u_H \in L_2(I;H^{-1}(\Omega ))$. Using $v \in W_\infty ^1(I;L_\infty (\Omega ))$ and $\partial _x u_H \in L_2(I \times \Omega )$, the first term is even in $L_2(I \times \Omega )$. Writing the second term as

$$\begin{aligned} (v(t,x)-v_{0} )\partial _t\partial _x u_H (t,x)={\textstyle \frac{v(t,x)-v(0,x)}{t}}t \partial _t\partial _x u_H (t,x)+{\textstyle \frac{v(0,x)-v_{0} }{x}}x \partial _t\partial _x u_H (t,x), \end{aligned}$$

from $t \partial _t\partial _x u_H \in L_2(I \times \Omega )$ and ${\textstyle \frac{v(t,x)-v(0,x)}{t}} \in L_\infty (I\times \Omega )$ by $v \in W^1_\infty (I;L_\infty (\Omega ))$, we have ${\textstyle \frac{v(t,x)-v(0,x)}{t}}t \partial _t\partial _x u_H (t,x) \in L_2(I\times \Omega )$. Similarly, from $x \partial _t\partial _x u_H (t,x) \in L_2(I;H^{-1}(\Omega ))$ and ${\textstyle \frac{v(0,x)-v_{0} }{x}} \in L_\infty (I;W^1_\infty (\Omega ))$ by $v \in L_\infty (I;W^2_\infty (\Omega ))$, we have ${\textstyle \frac{v(0,x)-v_{0} }{x}}x \partial _t\partial _x u_H (t,x)$ $\in$ $L_2(I;H^{-1}(\Omega ))$, so that $\partial _t g \in L_2(I;H^{-1}(\Omega ))$.

It remains to show that $g \in L_2(I;H^1(\Omega ))$. It is clear that $(v-v_{0} )\partial _x u_H \in L_2(I \times \Omega )$ and $(\partial _x v) \partial _x u_H \in L_2(I\times \Omega )$ by $v \in L_\infty (I;W_\infty ^1(\Omega ))$. Writing

$$\begin{aligned} (v(t,x)-v_{0} )\partial ^2_x u_H (t,x)={\textstyle \frac{v(t,x)-v(0,x)}{t}}t \partial ^2_x u_H (t,x)+{\textstyle \frac{v(0,x)-v_{0} }{x}}x \partial ^2_x u_H (t,x), \end{aligned}$$

from ${\textstyle \frac{v(t,x)-v(0,x)}{t}},\,{\textstyle \frac{v(0,x)-v_{0} }{x}}\in L_\infty (I \times \Omega )$ by $v \in W^1_\infty (I\times \Omega )$, and both $t \partial ^2_x u_H (t,x)$ and $x \partial ^2_x u_H (t,x) \in L_2(I\times \Omega )$, we obtain $g \in L_2(I;H^1(\Omega ))$, and the proof is completed. $\square$

By combining the results of the preceding three propositions with the regularity result proven in Proposition 3.1 we obtain the following.

#### Theorem 3.1

If $$v \in W^1_\infty (I \times \Omega ) \cap L_\infty (I;W^2_\infty (\Omega ))$$, then

$$\begin{aligned} e(v)\in H^1_{0,\{0\}}(I;H^1_0(\Omega ))\cap H^2(I;H^{-1}(\Omega )) \cap L_2(I;H^3(\Omega )). \end{aligned}$$

#### Proof

We obtain $(v-v_{0})\partial _x(u(v_{0})-u_H)$ $\in$ $L_2(I;H^1(\Omega ))$ $\cap$ $H^1(I;H^{-1}(\Omega ))$ from Lemma 3.2 and 3.3, whereas Lemma 3.4 implies that $(v-v_{0})\partial _x u_H \in L_2(I;H^1(\Omega )) \cap H^1(I;H^{-1}(\Omega ))$. We conclude that

$$(v-v_{0})\partial _xu(v_{0}) \in L_2(I;H^1(\Omega )) \cap H^1(I;H^{-1}(\Omega )),$$

so that an application of Proposition 3.1a) completes the proof. $\square$

Notice that as a consequence of Corollary 3.1, Lemma 3.2 and 3.3, $u(v_0)\notin H_{0,\{0\}}^1(I; L_2(\Omega ))\cup L_2(I;H^2(\Omega ))$. Comparing Corollary 3.1 with Theorem 3.1, we conclude that

$$\begin{aligned} e(v)=u(v)-u(v_0)~is indeed smoother than~ u(v_{0}), and thus than~ u(v), \end{aligned}$$

confirming the claim we made at the beginning of Section 3.

## Minimal residual method

For solving (3.3) (specifically for the forcing function *f* as in (3.6), i.e., for solving *e*(*v*)), we write it in variational form, i.e., we multiply it by test functions $z:I\times \Omega \rightarrow \mathbb R$ from a suitable collection, integrate it over $I \times \Omega$, and apply integration by parts with respect to *x*. We thus arrive at

$$\begin{aligned}&(B w)(z):=\\&\int _I \int _D \partial _t w(t,x) z(t,x)+\partial _x w(t,x) \partial _x z(t,x)-v(t,x)\partial _x w(t,x)z(t,x)\,dx\,dt\\&= \int _I \int _D f(t,x)z(t,x)\,dx\,dt=:f(z) \end{aligned}$$

for all those test functions. With

$$\begin{aligned} X:=L_2(I;H^1_0(\Omega )) \cap H^1(I;H^{-1}(\Omega )), \quad Y:=L_2(I;H^1_0(\Omega )), \end{aligned}$$

it is known that $(B,\gamma _0) \in \mathcal L_{\mathrm {iso}}(X ,Y'\times L_2(\Omega ))$, where $\gamma _0:= w \mapsto w(0,\cdot )$ denotes the initial trace operator, see, e.g., [31, Chapter IV] or [27].

Already because $X \ne Y \times L_2(\Omega )$, the well-posed system $(B,\gamma _0)w=(f,0)$ cannot be discretized by simple Galerkin discretizations. Given a family $(X_h)_{h \in \Delta }$ of finite dimensional subspaces of *X*, as discrete approximations to *w* one may consider the minimizers ${{\,\mathrm{argmin}\,}}_{\bar{w} \in X_h} \Vert B \bar{w}-f\Vert ^{2}_{Y'} +\Vert \gamma _0 \bar{w}\Vert ^{2}_{L_2(\Omega )}$. Since the dual norm $\Vert \cdot \Vert _{Y'}$ cannot be evaluated, this approach is not immediately feasible either. Therefore, for $(Y_h)_{h \in \Delta }$ being a second family of finite dimensional subspaces, now of *Y*, for $h \in \Delta$ as a discrete approximation from $X_h$ we consider

4.1 $$\begin{aligned} w_h:={{\,\mathrm{argmin}\,}}_{\bar{w} \in X_h} \Vert B \bar{w}-f\Vert ^2_{Y_h'} +\Vert \gamma _0\bar{w}\Vert ^{2}_{L_2(\Omega )}. \end{aligned}$$

This minimal residual approach has been studied for general parabolic PDEs in, e.g., [2, 28, 29], where $\Omega$ can be a *d*-dimensional spatial domain for arbitrary $d \ge 1$.

For parabolic differential operators with a possibly asymmetric spatial part, in our setting caused by a non-zero drift function *v*, in [28, Thm. 3.1] it has been shown that if $X_h \subset Y_h$ and

4.2 $$\begin{aligned} \varrho :=\inf _{h \in \Delta } \inf _{0 \ne \bar{w} \in X_h} \frac{\Vert \partial _t \bar{w}\Vert _{Y_h'}}{\Vert \partial _t \bar{w}\Vert _{Y'}}>0, \end{aligned}$$

then

4.3 $$\begin{aligned} \Vert w-w_h\Vert _X \lesssim \min _{\bar{w} \in X_h} \Vert w-\bar{w}\Vert _X, \end{aligned}$$

where the implied constant in (4.3) depends only on $\varrho$ and an upper bound for $\Vert v\Vert _{L_\infty (I \times \Omega )}$, i.e., $w_h$ is a *quasi-best approximation* from $X_h$ with respect to the norm on *X*.

### Remark 4.1

This quasi-optimality result has been demonstrated under the condition that the spatial part of the parabolic differential operator is *coercive* on $H^1_0(\Omega ) \times H^1_0(\Omega )$ for a.e. $t \in I$, i.e.,

$$\begin{aligned} \int _D \eta ' \eta '-v(t,\cdot ) \eta ' \eta \,dx \gtrsim \Vert \eta \Vert ^2_{H^1(\Omega )} \quad (\eta \in H_0^1(\Omega )), \end{aligned}$$

which holds true when $\partial _x v \le 0$ or $\Vert v\Vert _{L_\infty (I\times \Omega )} \sup _{0 \ne \eta \in H^1_0(\Omega )} \frac{\Vert \eta \Vert _{L_2(\Omega )}}{\Vert \eta '\Vert _{L_2(\Omega )}}<1$, but which might be violated otherwise.

Although this coercivity condition might not be necessary, it can always be enforced by considering $w_\lambda (t,\cdot ):=w(t,\cdot ) e^{-\lambda t}$, $f_\lambda (t,\cdot ):=f(t,\cdot ) e^{-\lambda t}$ instead of *w* and *f* with $\lambda$ sufficiently large, see also the proof of Proposition 3.1. By approximating $w_\lambda$ by the minimal residual method, and by multiplying the obtained approximation by $e^{\lambda t}$, an approximation for *w* is obtained. Since qualitatively the transformations with $e^{\pm \lambda t}$ do not affect the smoothness of solution or right-hand side, for convenience in the following we pretend that coercivity holds true for (3.3).

As in [28, 29], we equip $Y_h$ in (4.1) with the energy norm

$$\begin{aligned} \Vert z\Vert _Y^2 := (A_s z)(z) \quad (z\in Y_h), \end{aligned}$$

where

$$\begin{aligned}&(A_s z)(\bar{z}) :=\\&\int _I \int _D \partial _x z(t,x)\partial _x \bar{z} (t,x) - \frac{v(t,x)}{2} (\partial _x z(t,x) \bar{z}(t,x) + z(t,x) \partial _x \bar{z}(t,x)) \,d x\,dt \end{aligned}$$

denotes the symmetric part of the spatial differential operator. Equipping $Y_h$ and $X_h$ with bases $\Phi ^h=\{\phi ^h_i\}$ and $\Psi ^h=\{\psi ^h_j\}$, respectively, and denoting by $\varvec{w}^h$ the representation of the minimizer $w_h$ with respect to $\Psi _h$, $\varvec{w}^h$ is found as the second component of the solution of

4.4 $$\begin{aligned} \left[ \begin{array}{@{}cc@{}} \varvec{A}_s^h &{} \varvec{B}^h \\ {\varvec{B}^h}^\top &{} \varvec{C}^h \end{array}\right] \left[ \begin{array}{@{}c@{}} \varvec{\mu }^h \\ \varvec{w}^h \end{array}\right] = \left[ \begin{array}{@{}c@{}} \varvec{f}^h \\ 0\end{array}\right] , \end{aligned}$$

where $(\varvec{A}_s^h)_{i j}:= (A_s\phi ^h_j)(\phi ^h_i)$, $\varvec{B}^h_{i j}:=(B \psi ^h_j)(\phi ^h_i)$, $\varvec{C}^h_{i j}:= \int _D \psi ^h_j(0,x) \psi ^h_i(0,x) \, dx$, and $\varvec{f}^h_i:=f(\phi ^{h}_i)$. The operator $A_s$ can be replaced by any other spectrally equivalent operator on $Y_h$ without compromising the quasi-optimality result (4.3). We refer to [28, 29] for details.

Let $P_1$ be the set of polynomials of degree one. Taking for $n:=1/h \in \mathbb N$,

4.5 $$\begin{aligned} V_{x,h}&:=\big \{\eta \in H^1_0(\Omega ):\eta |_{((i-1)h,ih)} \in P_1 \text { for } i=1,\dots ,n \big \},\nonumber \\ V_{t,h}&:=\big \{\zeta \in H^1(I):\zeta |_{((i-1)hT,ihT)} \in P_1 \text { for } i=1,\dots ,n \big \},\nonumber \\ X_h&:=V_{t,h} \otimes V_{x,h}, \end{aligned}$$

it is known, cf. [29, Sect. 4], that condition (4.2) is satisfied for

4.6 $$\begin{aligned} Y_h:=\big \{\zeta \in L_2(I):\zeta |_{((i-1)hT,ihT)} \in P_1 \text { for } i=1,\dots ,n \big \} \otimes V_{x,h}, \end{aligned}$$

where obviously also $X_h \subset Y_h$.

Applying this approach for $f=(v-v_{0})\partial _x u(v_{0})$, in view of (4.3) the error of the obtained approximation for *e*(*v*) with respect to the *X*-norm can be bounded by the error of the best approximation from $X_h$. To bound the latter error we recall from Theorem 3.1 that for $v \in W^1_\infty (I \times \Omega ) \cap L_\infty (I;W^2_\infty (\Omega ))$, it holds that

$$\begin{aligned} e(v)\in \big (H^1_{0,\{0\}}(I) \otimes H^1_0(\Omega ) \big )\cap \big (H^2(I) \otimes H^{-1}(\Omega ) \big ) \cap \big (L_2(I) \otimes H^3(\Omega )\big ). \end{aligned}$$

With $Q_{x,h}$, $Q_{t,h}$ denoting the $L_2(\Omega )$- or $L_2(I)$-orthogonal projectors onto $V_{x,h}$ or $V_{t,h}$, respectively, $Q_{t,h} \otimes Q_{x,h}$ is a projector onto $X_h$. Writing

$$\begin{aligned} \mathrm {Id}- Q_{t,h} \otimes Q_{x,h}=( \mathrm {Id}-Q_{t,h}) \otimes Q_{x,h}+\mathrm {Id}\otimes (\mathrm {Id}- Q_{x,h}), \end{aligned}$$

and using that

$$\begin{aligned}&\Vert \mathrm {Id}- Q_{x,h}\Vert _{\mathcal L(H^1_0(\Omega )\cap H^2(\Omega ),H^1_0(\Omega ))} \lesssim h,\quad \Vert Q_{x,h}\Vert _{\mathcal L(H_0^1(\Omega ),H_0^1(\Omega ))} \lesssim 1,\\&\Vert \mathrm {Id}- Q_{t,h}\Vert _{\mathcal L(H^1(I),L_2(I))} \lesssim h, \quad \Vert \mathrm {Id}\Vert _{\mathcal L(L_2(I),L_2(I))} = 1 \end{aligned}$$

by standard interpolation estimates and uniform $H^1$-boundedness of these $L_2$-orthogonal projectors, see e.g. [5, §3], one infers that

$$\begin{aligned} \Vert \mathrm {Id}- Q_{t,h} \otimes Q_{x,h}\Vert _{\mathcal L((L_2(I) \otimes (H^1_0(\Omega )\cap H^2(\Omega )) ) \cap ( H^1(I) \otimes H^1_0(\Omega )),L_2(I) \otimes H^1_0(\Omega ))} \lesssim h. \end{aligned}$$

Similarly using that

$$\begin{aligned}&\Vert \mathrm {Id}- Q_{x,h}\Vert _{\mathcal L(L_2(\Omega ),H^{-1}(\Omega ))}=\Vert \mathrm {Id}- Q_{x,h}\Vert _{\mathcal L(H^1_0(\Omega ),L_2(\Omega ))} \lesssim h,\\&\Vert Q_{x,h}\Vert _{\mathcal L(H^{-1}(\Omega ),H^{-1}(\Omega ))} =\Vert Q_{x,h}\Vert _{\mathcal L(H_0^{1}(\Omega ),H_0^{1}(\Omega ))} \lesssim 1, \\&\Vert \mathrm {Id}- Q_{t,h}\Vert _{\mathcal L(H^2(I),H^1(I))} \lesssim h, \quad \Vert \mathrm {Id}\Vert _{\mathcal L(H^1(I),H^1(I))} =1, \end{aligned}$$

one infers that

$$\begin{aligned} \Vert \mathrm {Id}- Q_{t,h} \otimes Q_{x,h}\Vert _{\mathcal L(( H^1(I) \otimes L_2(\Omega ) )\cap ( H^2(I) \otimes H^{-1}(\Omega ) ), H^1(I) \otimes H^{-1}(\Omega ))}\lesssim h. \end{aligned}$$

Our findings are summarized in the following theorem.

### Theorem 4.1

For $v \in W^1_\infty (I \times \Omega ) \cap L_\infty (I;W^2_\infty (\Omega ))$ and $X_h$, $Y_h$ as defined in (4.5) and (4.6), the numerical approximation $e_h=e_h(v) \in X_h$ to $e=e(v)$ obtained by the application of the minimal residual method to (3.1)^1^ satisfies

$$\begin{aligned} \Vert e-e_h\Vert _X \lesssim h. \end{aligned}$$

Notice that for this space $X_h$ of continuous piecewise bilinears, this linear decay of the error $\Vert e-e_h\Vert _X$ as function of *h* is generally the best that can be expected. In view of the order of the space $X_h$, one may hope that $\Vert e-e_h\Vert _{L_2(I \times \Omega )}$ is ${\mathcal O}(h^2)$, but on the basis of the smoothness demonstrated for *e*, even for $\inf _{\bar{e} \in X_h}\Vert e-\bar{e}\Vert _{L_2(I \times \Omega )}$ this cannot be shown.

## Interpolation for parametrized drift, boundaries, and final time

In this section we consider the case that *v* and *T* in (2.3) depend on a number of parameters $(\rho _1,\ldots ,\rho _N) \in [-1,1]^N$, and that one is interested in the solution *u*(*v*) to (2.3) for multiple values of these parameters. As explained in Section 3, in order to find *u*(*v*) it suffices to obtain the solution *e*(*v*) to (3.1). Instead of simply solving *e*(*v*) for each of the desired parameter values, under the provision that *v* and *T* depend smoothly on the parameters, one may attempt to *interpolate* *e*(*v*) from its a priori computed approximations for a carefully selected set of parameters in $[-1,1]^N$.

In order to be able to do so, first of all we need to get rid of the parameter dependence of the domain $I \times \Omega =(0,T) \times (0,1)$. With $\hat{I}:=(0,1)$, the function $\hat{u}$ on $\hat{I}\times \Omega$ defined by $\hat{u}(t,x):=u(t T,x)$ solves

5.1 $$\begin{aligned} \left\{ \begin{aligned} \partial _t \hat{u}(t, x)&=T[\partial ^2_x \hat{u}(t, x) +\hat{v}(t,x) \partial _x \hat{u}(t, x)]&\quad (t, x) \in \hat{I} \times D,\\ \hat{u}(t,0)&=1, \quad \hat{u}(t,1)=0&\quad t \in \hat{I},\\ \hat{u}(0, x)&=0&\quad x \in D, \end{aligned} \right. \end{aligned}$$

where analogously $\hat{v}(t,x):=v(t T,x)$. Denoting this $\hat{u}$ as $\hat{u}(\hat{v},T)$, the difference

$$\begin{aligned} \hat{e}=\hat{e}(\hat{v},T):=\hat{u}(\hat{v},T)-\hat{u}(v_{0},T):(t,x) \mapsto e(tT,x) \end{aligned}$$

solves

5.2 $$\begin{aligned} \left\{ \begin{aligned} \partial _t \hat{e}(t, x)&= T[\partial ^2_x \hat{e}(t, x) + \hat{v}(t,x) \partial _x \hat{e}(t, x)] \\&\quad +T(\hat{v}(t,x)-v_{0}) \partial _x \hat{u}(v_{0},T)&\quad (t, x) \in \hat{I} \times \Omega ,\\ \hat{e}(t,0)&=0, \quad \hat{e}(t,1)=0&\quad t \in \hat{I},\\ \hat{e}(0, x)&=0&\quad x \in \Omega . \end{aligned} \right. \end{aligned}$$

By simply replacing $I=(0,T)$ by $\hat{I}=(0,1)$ and in particular *X* as well as *Y* by

$$\begin{aligned} \hat{X}:=L_2(\hat{I};H^1_0(\Omega )) \cap H^1(\hat{I};H^{-1}(\Omega )), \quad \hat{Y}:=L_2(\hat{I};H^1_0(\Omega )), \end{aligned}$$

in a number of places, it is clear that the results that we obtained about the smoothness of *e* and its numerical approximation $e_h$ by the minimal residual method apply equally well to $\hat{e}$ and its minimal residual approximation that we denote as $\hat{e}_h$.

Since the domain of $\hat{e}$ is independent of parameters, we can apply the idea of interpolation. One option is to perform a ‘full’ tensor product interpolation. In this case, the number of interpolation points required for a fixed polynomial degree, i.e., the number of values of the parameters for which a numerical approximation for $\hat{e} \in \hat{X}$ has to be computed, grows exponentially with the number *N* of parameters. As this is undesirable, we instead apply a sparse tensor product interpolation. More specifically, we choose the Smolyak construction, based on Clenshaw–Curtis abscissae in each parameter direction, see [22]: For $i\in \mathbb N$ let $I_{i+1}$ denote the univariate interpolation operator with abscissae $\cos j 2^{-i} \pi$, $j=0,\ldots ,2^i$, onto the space of polynomials of degree $2^i$, let $I_1$ be the interpolation operator with abscissa 0 and let $I_{0}:=0$. Then, for an integer $q\ge N$, we apply the sparse interpolator

$$\begin{aligned} {\mathcal {I}}_q:=\sum _{\{\mathbf{i} \in \mathbb N_0^N:\sum _{n=1}^N i_n \le q\}} \bigotimes _{n=1}^N (I_{i_n}-I_{i_n-1}). \end{aligned}$$

It is known that the resulting interpolation error in $C([-1,1]^N;\hat{X})$ (for arbitrary Banach space $\hat{X}$), equipped with $\Vert \cdot \Vert _{L_\infty ([-1,1]^N;\hat{X})}$, decays subexponentially in the number of interpolation points when $\hat{e}$ as function of each of the parameters $\rho _n$ has an extension to a differentiable mapping on a neighbourhood $\Sigma$ of $[-1,1]$ in $\mathbb C$. For details about this statement we refer to [22, Thm. 3.11]. [22] also mentions that the result requires relatively large values of *q*. Thus, the authors additionally prove algebraic convergence under the same assumptions but for arbitrary *q* [22, Thm. 3.10].

Instead of $\hat{e}$, we interpolate a numerical approximation $\hat{e}_h$, specifically the one obtained by the minimal residual method described in Section 4. For the additional error we have

$$\begin{aligned} \Vert {\mathcal {I}}_q(\hat{e}-\hat{e}_h)\Vert _{L_\infty ([-1,1]^N;\hat{X})} \le \Vert {\mathcal {I}}_q \Vert _{\mathcal L(C([-1,1]^N),C([-1,1]^N))} \Vert \hat{e}-\hat{e}_h\Vert _{L_\infty ([-1,1]^N;\hat{X})}. \end{aligned}$$

In [8, Sect. 5.3] it has been shown that the factor $\Vert {\mathcal {I}}_q\Vert _{\mathcal L(C([-1,1]^N),C([-1,1]^N))}$, known as the Lebesgue constant, is bounded by $(\# \{\mathbf{i} \in \mathbb N_0^N:\sum _{n=1}^N i_n \le q\})^2$, which is only of polylogarithmic order as function of the number of interpolation points.

Concerning the factor $\Vert \hat{e}-\hat{e}_h\Vert _{L_\infty ([-1,1]^N;\hat{X})}$, in our derivation of Theorem 4.1 we have seen that for each parameter value $(\rho _1,\ldots ,\rho _N) \in [-1,1]^N$ the expression $h^{-1}\Vert \hat{e}-\hat{e}_h\Vert _{\hat{X}}$ can be bounded by a constant multiple, only dependent on an upper bound for $\Vert \hat{v}\Vert _{L_\infty (\hat{I} \times \Omega )}$ and for the norm of $\hat{e}$ in $H^1_{0,\{0\}}(\hat{I};H^1_0(\Omega ))\cap H^2(\hat{I};H^{-1}(\Omega )) \cap L_2(\hat{I};H^2(\Omega ))$. For uniformly bounded *T* and $T^{-1}$, and $\hat{v}$ that varies over a bounded set in $W^1_\infty (\hat{I} \times \Omega ) \cap L_\infty (\hat{I};W^2_\infty (\Omega ))$, inspection of the estimates from Sect. 3 reveals that the latter norm of $\hat{e}$ is uniformly bounded. So assuming that these conditions on *T*, $T^{-1}$ and *v* hold true for $(\rho _1,\ldots ,\rho _N) \in [-1,1]^N$, we have that $\Vert \hat{e}-\hat{e}_h\Vert _{L_\infty ([-1,1]^N;\hat{X})} \lesssim h$.

What remains is to establish the differentiability of the solution $\hat{e}$ as function of each of the parameters which is done in the following theorem.

### Theorem 5.1

For an open $[-1,1] \subset \Sigma \subset \mathbb C$, let $(\hat{v},T):\Sigma \rightarrow C(\overline{\hat{I}};W^1_\infty (\Omega )) \times (0,\infty )$ be differentiable. For $\rho \in \Sigma$ let $\hat{e}(\hat{v}(\rho ),T(\rho ))\in \hat{X}$ be the solution to (5.2). Then $\rho \mapsto \hat{e}=\hat{e}(\hat{v}(\rho ),T(\rho )):\Sigma \rightarrow \hat{X}$ is differentiable.

### Proof

The proof is based on the fact that $\hat{e}$ is the solution of a well-posed PDE with coefficients and a forcing term that are differentiable functions of $\rho$.

Analogously to (3.4), denoting by $L(\hat{v},T)$ the map $w \mapsto f$ defined by $\partial _t w=T(\partial _x^2+\hat{v} \partial _x) w+f$ on $\hat{I} \times \Omega$, $w(t,0)=0=w(t,1)$ ($t \in \hat{I}$), and $w(0,x)=0$ ($x \in \Omega$), one has

5.3 $$\begin{aligned} \hat{e}(\hat{v}(\rho ),T(\rho ))=L(\hat{v}(\rho ),T(\rho ))^{-1} T(\rho )(\hat{v}(\rho )-v_{0}(\rho ))\partial _x \hat{u}(v_{0}(\rho ),T(\rho )), \end{aligned}$$

where $v_{0}(\rho ):=\hat{v}(\rho )(0,0)$. Below we demonstrate that

5.4 $$\begin{aligned}&\rho \mapsto T(\rho )(\hat{v}(\rho )-v_{0}(\rho )):\Sigma \rightarrow L_\infty (\hat{I};W_\infty ^1(\Omega )) \text { is differentiable,} \end{aligned}$$

5.5 $$\begin{aligned}&\rho \mapsto \hat{u}(v_{0}(\rho ),T(\rho )):\Sigma \rightarrow L_2(\hat{I} \times \Omega ) \text { is differentiable,} \end{aligned}$$

so that, from $\partial _x \in \mathcal L(L_2(\hat{I}\times \Omega ),\hat{Y}')$ and $L_\infty (\hat{I};W_\infty ^1(\Omega ))$-functions being pointwise multipliers in $\mathcal L(\hat{Y}',\hat{Y}')$,

5.6 $$\begin{aligned}&\rho \mapsto T(\rho ) (\hat{v}(\rho )-v_{0}(\rho ))\partial _x \hat{u}(v_{0}(\rho ),T(\rho )):\Sigma \rightarrow \hat{Y}' \text { is differentiable.} \end{aligned}$$

We proceed below to show that

5.7 $$\begin{aligned}&\rho \mapsto L(\hat{v}(\rho ),T(\rho ))^{-1}:\Sigma \rightarrow \mathcal L(\hat{Y}',\hat{X}) \text { is differentiable.} \end{aligned}$$

Together, (5.6) and (5.7) complete the proof.

From $\rho \mapsto \hat{v}(\rho ) :\Sigma \rightarrow C(\overline{\hat{I}};W^1_\infty (\Omega ))$ being differentiable, it follows that $\rho \mapsto v_{0}(\rho ) :\Sigma \rightarrow \mathbb C$ is differentiable, which together with $T:\Sigma \rightarrow (0,\infty )$ being differentiable shows (5.4).

To show (5.7), we fix some arbitrary $\rho _0\in \Sigma$, abbreviate $L:=L(\hat{v}(\rho ),T(\rho ))$ as well as $L_0:=L(\hat{v}(\rho _0),T(\rho _0))$ and write

$$\begin{aligned} L^{-1}=L_0^{-1}+L_0^{-1}[L_0-L] L_0^{-1}+L^{-1}\{[L_0-L] L_0^{-1}\}^2. \end{aligned}$$

This decomposition and the fact that $L(\hat{v}(\rho ),T(\rho ))^{-1}$ is bounded in $\mathcal L(\hat{Y}',\hat{X})$ for $\rho$ in a neighbourhood of $\rho _0$ ([27, Thm. 5.1]) imply that it suffices to show that for some $K(\rho _0) \in \mathcal L(\mathbb C,\mathcal L(\hat{X},\hat{Y}'))$,

5.8 $$\begin{aligned} L(\hat{v}(\rho _0),T(\rho _0))-L(\hat{v}(\rho ),T(\rho ))=K(\rho _0) (\rho -\rho _0)+o(\rho -\rho _0) \text { in }\mathcal L(\hat{X},\hat{Y}'). \end{aligned}$$

We have

$$\begin{aligned}&L(\hat{v}(\rho _0),T(\rho _0))-L(\hat{v}(\rho ),T(\rho ))\\&=[T(\rho )-T(\rho _0)]\partial _x^2 + [(T(\rho )-T(\rho _0))\hat{v}(\rho )+T(\rho _0)(\hat{v}(\rho )-\hat{v}(\rho _0))]\partial _x. \end{aligned}$$

From $T(\rho )-T(\rho _0)=DT(\rho _0)(\rho -\rho _0)+o(\rho -\rho _0)$, $\hat{v}(\rho )-\hat{v}(\rho _0)=D\hat{v}(\rho _0)(\rho -\rho _0)+o(\rho -\rho _0)$ in $C(\overline{I}_1,W_\infty ^1(\Omega )) \hookrightarrow L_\infty (\hat{I} \times \Omega )$, $\partial _x^2 \in \mathcal L(\hat{X},\hat{Y}')$, $\partial _x \in \mathcal L(\hat{X},L_2(\hat{I} \times \Omega ))$, $L_\infty (\hat{I} \times \Omega )$-functions being pointwise multipliers in $\mathcal L(L_2(\hat{I} \times \Omega ),L_2(\hat{I} \times \Omega ))$, and $L_2(\hat{I} \times \Omega ) \hookrightarrow \hat{Y}'$, one concludes (5.8), and so (5.7).

To show (5.5), i.e., differentiability of $\rho \mapsto \hat{u}(v_{0}(\rho ),T(\rho ))$, we repeat the argument that led to (5.3) to obtain

$$\begin{aligned} \hat{u}(v_{0}(\rho ),T(\rho ))&=\hat{u}(0,T(\rho ))+\hat{u}(v_{0}(\rho ),T(\rho ))-\hat{u}(0,T(\rho ))\\&=\hat{u}(0,T(\rho ))+T(\rho )v_{0}(\rho ) L(v_{0}(\rho ),T(\rho ))^{-1} \partial _x \hat{u}(0,T(\rho )), \end{aligned}$$

and show that

5.9 $$\begin{aligned} \rho \mapsto \hat{u}(0,T(\rho )):\Sigma \mapsto L_2(\hat{I} \times \Omega ) \text { is differentiable.} \end{aligned}$$

Then $\rho \mapsto \partial _x\hat{u}(0,T(\rho )):\Sigma \mapsto \hat{Y}'$ is differentiable, and from both $\rho \mapsto T(\rho )v_{0}(\rho ):$ $\Sigma \rightarrow \mathbb C$ and $\rho \mapsto L(v_{0}(\rho ),T(\rho ))^{-1}:\Sigma \rightarrow \mathcal L(\hat{Y}',\hat{X})$ being differentiable one infers (5.5).

To show (5.9), we apply our approach for the third time. Picking some $\bar{\rho } \in \Sigma$, we write

$$\begin{aligned} \hat{u}(0,T(\rho ))=\hat{u}(0,T(\bar{\rho } ))+(T(\bar{\rho })-T(\rho ))L(0,T(\rho ))^{-1} \partial _x^2 \hat{u}(0,T(\bar{\rho } )). \end{aligned}$$

Knowing that $\partial _x^2 \hat{u}(0,T(\bar{\rho } )) \in \hat{Y}'$, and $\rho \mapsto L(0,T(\rho ))^{-1}:\Sigma \rightarrow \mathcal L(\hat{Y}',\hat{X})$ and $\rho \mapsto T(\rho ) :\Sigma \rightarrow \mathbb C$ are differentiable, the proof of (5.9) and thus of the theorem is completed. $\square$

## Numerical results

We consider three relevant examples of the form (1.3) (or its equivalent reformulation (2.1)) with $\sigma =1$ from the literature. We transform the solution $\tilde{u}$ of (2.1), which might live on a time-dependent spatial domain, to *u*, which satisfies (2.3) on the domain $(0,T) \times (0,1)$. In each example the resulting drift function *v* as well as the end time point *T* depend on an up to $N=5$ dimensional parameter ${\varvec{\rho }} \in [-1,1]^N$.

As $u(v({\varvec{\rho }})(0,0),T({\varvec{\rho }}))$ can be computed efficiently as a truncated series, it suffices to consider the difference

$$\begin{aligned} e(v({\varvec{\rho }}),T({\varvec{\rho }}))=u(v({\varvec{\rho }}),T({\varvec{\rho }}))-u(v({\varvec{\rho }})(0,0),T({\varvec{\rho }})), \end{aligned}$$

which satisfies equation (3.1) and is provably smoother than *u* (Theorem 3.1).

Thinking of a multi-query setting, instead of approximating this difference for each individual parameter value of interest we want to use (sparse) interpolation in the parameter domain $[-1,1]^N$. To that end, defining $\hat{e}(t,x):=e(tT({\varvec{\rho }}),x)$, we get rid of the parameter-dependent domain $(0,T({\varvec{\rho }}))\times \Omega$ on which *e* lives. This function $\hat{e}(t,x)$ satisfies the parabolic problem equation (5.2) on the space-time domain $\hat{I}\times \Omega =(0,1)^2$ with forcing term

$$\begin{aligned} \bar{w}&\mapsto \int _0^1\int _D (\hat{v}(t,x) - v_{0})\partial _x \hat{u}(v_{0})(t,x) \bar{w}(t,x) \,dx\,dt\\&=\int _0^1\int _D \hat{u}(v_{0})(t,x) \big (-\partial _x\hat{v}(t,x) \bar{w}(t,x) - (\hat{v}(t,x)-v_0) \partial _x\bar{w}(t,x)\big )\,dx\,dt \end{aligned}$$

for all $\bar{w}\in \hat{X}=L_2(\hat{I};H^1_0(\Omega )) \cap H^1(\hat{I};H^{-1}(\Omega ))$, and $v_0:=v({\varvec{\rho }})(0,0)$ and corresponding $\hat{u}(v_0)$ solving (5.1) with $\hat{v} = v_0$.

For all sparse interpolation points, by applying the minimal residual method from Section 4 we approximate $\hat{e}$ by the continuous piecewise affine function $\hat{e}_h$ on a uniform tensor mesh with mesh-size *h*, where $\hat{u}(v_{0})$ inside the forcing term can be efficiently approximated at high accuracy as a truncated series.

Finally, for all parameter values $\varvec{\rho }$ of interest, we apply the sparse tensor product interpolation analyzed in Section 5 giving rise to an overall error

$$\begin{aligned} \Vert \hat{e} - \mathcal {I}_q\hat{e}_h\Vert _{\hat{X}} \le \Vert \hat{e} - \hat{e}_h\Vert _{\hat{X}} + \Vert \hat{e}_h - \mathcal {I}_q \hat{e}_h\Vert _{\hat{X}} \approx \Vert \hat{e}_{h/2} - \hat{e}_h\Vert _{\hat{X}} + \Vert \hat{e}_h - \mathcal {I}_q \hat{e}_h\Vert _{\hat{X}} \end{aligned}$$

with *q* the parameter that steers the accuracy of the sparse interpolation. For each of the considered three examples, we compute the latter two errors for different *h* and *q* and parameter test set

6.1 $$\begin{aligned} \varvec{\rho } \in \{-1,-0.5,0.5,1\}^N. \end{aligned}$$

By Theorem 4.1, we expect $\Vert \hat{e}_{h/2} - \hat{e}_h\Vert _{\hat{X}} = \mathcal {O}(h)$ for the first term. Section 5 suggests subexponential convergence of the second term $\Vert \hat{e}_h - \mathcal {I}_q \hat{e}_h\Vert _{\hat{X}}$ as function of the number of interpolation points (this was shown for $\Vert \hat{e} - \mathcal {I}_q \hat{e}\Vert _{\hat{X}}$). However, we already mentioned there that subexponential convergence is only observed for very high *q* and in practice one should rather expect algebraic convergence.

Notice that $\Vert \cdot \Vert _{\hat{X}}$ involves a negative order Sobolev norm. Thus, we compute an equivalent version of $\Vert \cdot \Vert _{\hat{X}}$ for functions in the discrete trial space $\bar{w}\in \hat{X}_h\subset \hat{X}$ (similarly for $\bar{w}\in \hat{X}_{h/2}$) (see [28, Proof of Thm. 3.1])

6.2 $$\begin{aligned} \Vert \bar{w} \Vert _{\hat{X}}^2 \eqsim (\bar{\varvec{w}}^{h})^\top (\varvec{B}^{h})^\top (\varvec{A}^{h})^{-1} \varvec{B}^{h} \bar{\varvec{w}}^{h} + (\bar{\varvec{w}}^{h})^\top \varvec{C}^{h} \bar{\varvec{w}}^{h}. \end{aligned}$$

Here, $\bar{\varvec{w}}^{h}$ is the coefficient vector of $\bar{w}$ in the standard nodal basis $\Psi ^{h}=\{\psi ^h_i\}$, $\varvec{B}^{h}$ and $\varvec{C}^{h}$ are defined as in (4.4) with the standard nodal basis $\Phi ^{h}=\{\phi ^{h}_i\}$, and $\varvec{A}^{h}_{i j}:=\int _I \int _\Omega \partial _x \phi ^{h}_j(t,x)\partial _x \phi ^{h}_i (t,x)\,d x\,dt$.

### Time-dependent hyperbolic drift function

As in [9, 19], we consider

$$\begin{aligned} \mu (t,x):= \mu _0 + \mu _1 \frac{t}{t+t_0} \end{aligned}$$

from Section 1 with parameters $\mu _0,\mu _1\in \mathbb R$ and $t_0>0$. The left and right boundary are given as

$$\begin{aligned} \alpha (t):=0\quad \text {and}\quad \beta (t):=\beta _0 \end{aligned}$$

with parameter $\beta _0>0$. Following [9, 19], we particularly consider the following practical ranges: $\mu _0 \in [-1.97,-1.64]$, $\mu _1\in [-2.31,-0.99]$, $t_0\in [0.13,0.40]$, $\beta _0\in [1.38,2.26]$, and $\tau \in [0.1,2.5]$ for the end-time point. We have $N=5$ different parameters on which $\tilde{v}$ and thus *v* depend. After rescaling, the parameter space hence has the form $[-1,1]^5$.

**Fig. 1:**
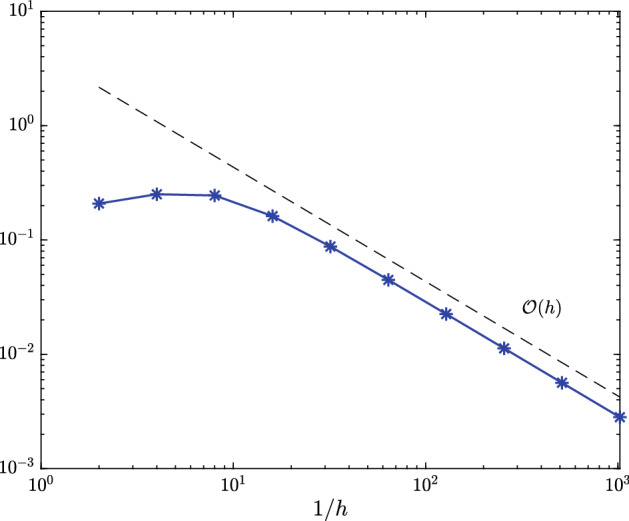
Maximal error $\hat{e}_{h/2}(\hat{v}({\varvec{\rho }})) - \hat{e}_h(\hat{v}({\varvec{\rho }}))$ measured in (equivalent) $\hat{X}$-norm over all ${\varvec{\rho }}\in \{-1,-0.5,0,0.5,1\}^5$ for time-dependent hyperbolic drift function from Sect. 6.1

**Fig. 2:**
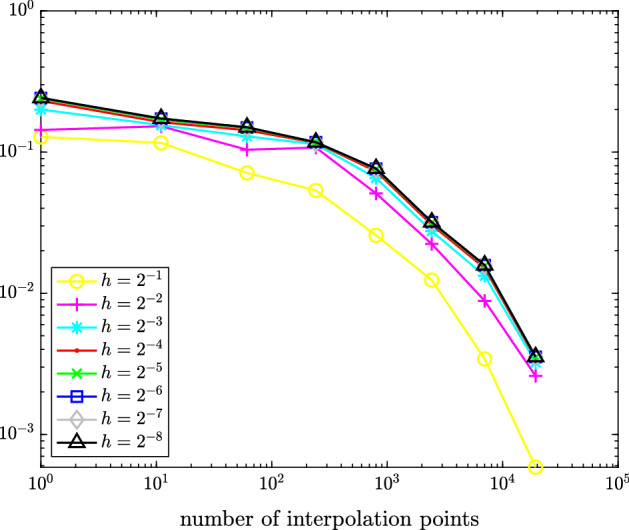
Maximal interpolation error $\hat{e}_{h}(\hat{v}({\varvec{\rho }})) - (\mathcal {I}_q\hat{e}_h(\hat{v}(\cdot )))({\varvec{\rho }})$ for various choices of *h* measured in (equivalent) $\hat{X}$-norm over all ${\varvec{\rho }}\in \{-1,-0.5,0,0.5,1\}^5$ for time-dependent hyperbolic drift function from Sect. 6.1

In Figure 1, we plot the maximal error $\hat{e}_{h/2} - \hat{e}_h\approx \hat{e} - \hat{e}_h$ measured in the (equivalent) $\hat{X}$-norm (6.2) over the test set (6.1) for different values of *h*. Figure 2 depicts the maximal interpolation error $\hat{e}_h - \mathcal {I}_q\hat{e}_h$ over the test set (6.1) for different values of *h* and *q*.

**Fig. 3:**
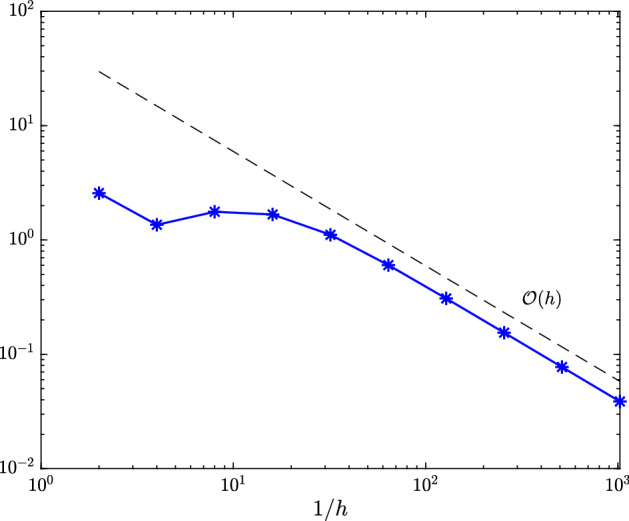
Maximal error $\hat{e}_{h/2}(\hat{v}({\varvec{\rho }})) - \hat{e}_h(\hat{v}({\varvec{\rho }}))$ measured in (equivalent) $\hat{X}$-norm over all ${\varvec{\rho }}\in \{-1,-0.5,0,0.5,1\}^3$ for space-dependent linear drift function from Sect. 6.2

**Fig. 4:**
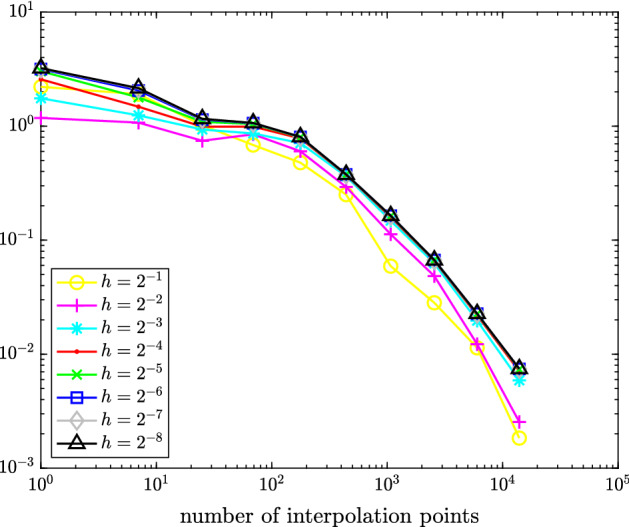
Maximal interpolation error $\hat{e}_{h}(\hat{v}({\varvec{\rho }})) - (\mathcal {I}_q\hat{e}_h(\hat{v}(\cdot )))({\varvec{\rho }})$ with *h* $=$ $2^{-1},\dots ,2^{-8}$ measured in (equivalent) $\hat{X}$-norm over all ${\varvec{\rho }}\in \{-1,-0.5,0,0.5,1\}^3$ for space-dependent linear drift function from Sect. 6.2

**Fig. 5:**
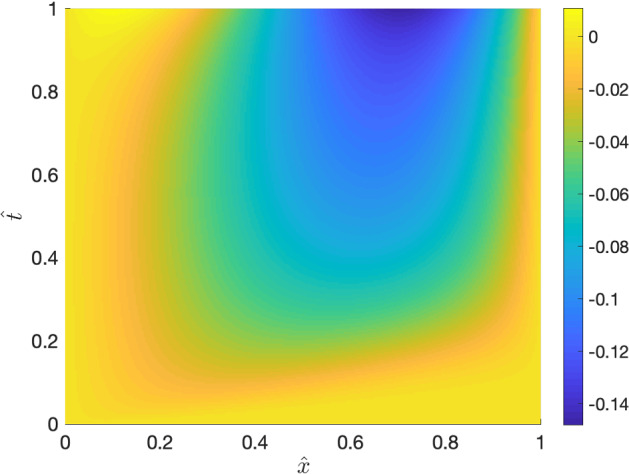
An approximation of solution $\hat{e}$ to (5.2) using the minimal residual method

**Fig. 6:**
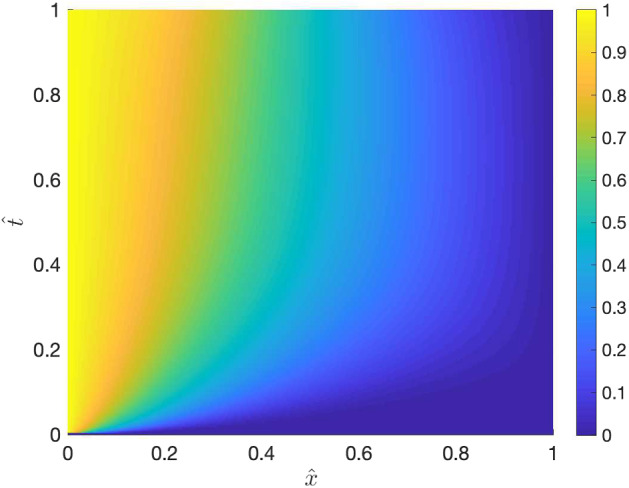
An approximation of the solution $\hat{u}$ to (5.1) obtained by adding $\hat{u}(v_0,T)$ to $\hat{e}$

**Fig. 7:**
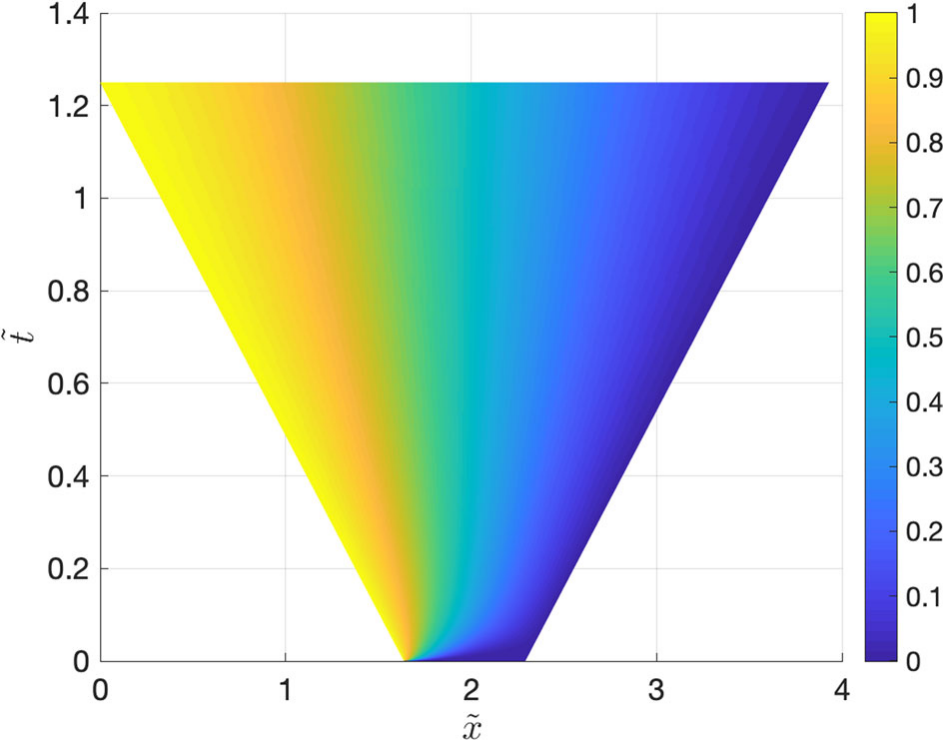
An approximation of the solution $\tilde{u}$ to (2.1) obtained by transforming the approximation of $\hat{u}$ back to the original domain

**Fig. 8:**
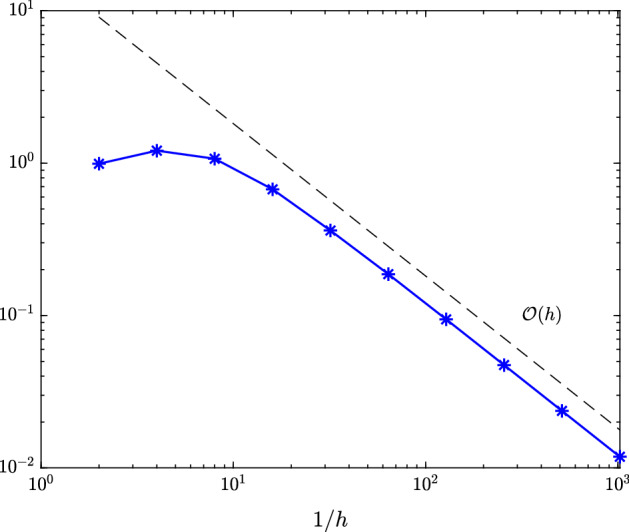
Maximal error $\hat{e}_{h/2}(\hat{v}({\varvec{\rho }}),T({\varvec{\rho }})) - \hat{e}_h(\hat{v}({\varvec{\rho }}),T({\varvec{\rho }}))$ measured in (equivalent) $\hat{X}$-norm over all ${\varvec{\rho }}\in \{-1,-0.5,0,0.5,1\}^4$ for constant drift function with time-dependent linear spatial domain from Sect. 6.3

**Fig. 9:**
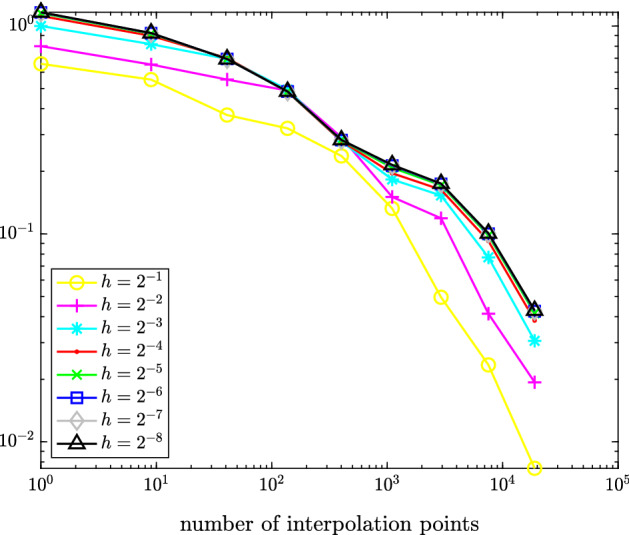
Maximal interpolation error $\hat{e}_{h}(\hat{v}({\varvec{\rho }}), T({\varvec{\rho }})) - (\mathcal {I}_q\hat{e}_h(\hat{v}(\cdot ),T(\cdot )))({\varvec{\rho }})$ for various choices of *h* measured in (equivalent) $\hat{X}$-norm over all ${\varvec{\rho }}\in \{-1,-0.5,0,0.5,1\}^4$ for constant drift function with time-dependent linear spatial domain from Sect. 6.3

### Space-dependent linear drift function

As in [26], we consider

$$\begin{aligned} \mu (t,x):= \mu _0 + \mu _1 (\beta _0 - x) \end{aligned}$$

from Section 1 with parameters $\beta _0>0$ and $\mu _0, \mu _1 \in \mathbb R$. The left and right boundary are again given as

$$\begin{aligned} \alpha (t):=0\quad \text {and}\quad \beta (t):=\beta _0. \end{aligned}$$

Motivated by [21, 26], we particularly consider the following practical ranges: $\mu _0 \in [-2,2]$, $\mu _1\in [-4,4]$, and $\beta _0 \in [0.5,2]$, and choose the end-time point as $\tau :=2.5$. We have $N=3$ different parameters on which $\tilde{v}$ and thus *v* depend. After rescaling, the parameter space hence has the form $[-1,1]^3$.

In Figure 3, we plot the maximal error $\hat{e}_{h/2} - \hat{e}_h\approx \hat{e} - \hat{e}_h$ measured in the (equivalent) $\hat{X}$-norm (6.2) over the test set (6.1). Figure 4 depicts the maximal interpolation error $\hat{e}_h - \mathcal {I}_q\hat{e}_h$ over the test set (6.1) for different values of *h* and *q*.

### Constant drift function and time-dependent linear spatial domain

We consider a constant drift function

$$\begin{aligned} \mu (t,x):= \mu _0 \end{aligned}$$

with parameter $\mu _0\in \mathbb R$. As in [13] (see also Example 2.1), we choose the left and right boundary as

$$\begin{aligned} \alpha (t):=\beta _0\frac{t}{2 T_0}\quad \text {and}\quad \beta (t):=\beta _0 \Big (1-\frac{t}{2 T_0}\Big ) \end{aligned}$$

with parameters $\beta _0,T_0>0$. Recall from Example 2.1 that

$$\begin{aligned} \theta (t) = \frac{ \beta _0^2(T_0-2\widetilde{T})^2 t }{ T_0^2 - 2\beta _0^2(T_0-2\widetilde{T})t },\quad t\in [0,T) \end{aligned}$$

with $T=\theta ^{-1}(\widetilde{T})=\frac{T_0 \widetilde{T}}{\beta _0^2 (T_0-2\widetilde{T})}$. Following [13], we particularly consider the following practical ranges: $\mu _0\in [-5.86,0]$, $\beta _0\in [0.56,3.93]$, $T_0 \in [3,20]$, and $\tau \in [0.1,2.5]$ for the end-time point. We have $N=4$ different parameters on which $\tilde{v}$ and thus *v* depend. After rescaling, the parameter space hence has the form $[-1,1]^4$. Figures 5, 6, and 7 show approximations of the solution $\hat{e}$ to (5.2), the solution $\hat{u}$ to (5.1), and the solution $\tilde{u}$ to the original problem (2.1), with parameter values $\mu _0 = 0$, $\beta _0 = 3.93$, $T_0 = 3$, and $\tau = 2.5$. In Figure 8, we plot the maximal error $\hat{e}_{h/2} - \hat{e}_h\approx \hat{e} - \hat{e}_h$ measured in the (equivalent) $\hat{X}$-norm (6.2) over the test set (6.1). Figure 9 depicts the maximal interpolation error $\hat{e}_h - \mathcal {I}_q\hat{e}_h$ over the test set (6.1) for different values of *h* and *q*.

## Conclusion

We have developed a numerical solution method for solving the Fokker–Planck equation on a one-dimensional spatial domain and with a discontinuity between initial and boundary data and time-dependent boundaries. We first transformed the equation to an equation on a rectangular time-space domain. We then demonstrated that the solution of a corresponding equation with a suitable constant drift function, whose solution is explicitly available as a fast converging series expansion, captures the main singularity present in the solution for a variable drift function. The equation for the difference of both these solutions, which is thus more regular than both terms, is solved with a minimal residual method. This method is known to give a quasi-best approximation from the selected trial space.

Finally, in order to efficiently solve Fokker–Planck equations that depend on multiple parameters, we demonstrate that the solution is a holomorphic function of these parameters. Consequently, a sparse tensor product interpolation method can be shown to converge at a subexponentional rate as function of the number of interpolation points. In one test example, this interpolation method works very satisfactory, but the results are less convincing in two other cases. We envisage that in those cases better results can be obtained by an adaptive sparse interpolation method as the one proposed in [6].

## Footnotes

1 If necessary taking into account the transformations discussed in Remark 4.1.
